# Influence and mechanism of sodium-glucose cotransporter-2 inhibitors on the cardiac function: study protocol for a prospective cohort study

**DOI:** 10.3389/fendo.2023.1199960

**Published:** 2023-07-19

**Authors:** Min-Jia Cao, Fang-Hong Shi, Bin-Bin Yu, Xue-Chen Ma, Chen Zhang, Li Xu, Yi-Hong Jiang, Heng Ge, Long Shen, Jun Pu

**Affiliations:** ^1^ Department of Pharmacy, Ren Ji Hospital, Shanghai Jiao Tong University School of Medicine, Shanghai, China; ^2^ Department of Pharmacy, Huangyan Hospital of Wenzhou Medical University, Taizhou First People’s Hospital, Zhejiang, China; ^3^ Department of Pharmacy, The Third People’s Hospital of Xining, Xining, China; ^4^ Department of Cardiology, Ren Ji Hospital, Shanghai Jiao Tong University School of Medicine, Shanghai, China; ^5^ Department of Endocrinology, Ren Ji Hospital, Shanghai Jiao Tong University School of Medicine, Shanghai, China

**Keywords:** cardiac function, cardiac mechanisms, SGLT2 inhibitors, prospective cohort study without control, diabetes

## Abstract

**Background:**

Acute myocardial infarction (AMI) poses a significant threat to cardiovascular diseases (CVDs), leading to a high risk of heart failure (HF) and cardiovascular death. Growing evidence has unveiled the potential of sodium-glucose cotransporter-2 (SGLT2) inhibitors to improve cardiovascular outcomes in patients with CVD regardless of diabetes, but there is limited evidence in AMI patients. Furthermore, it is controversial whether the effects can be ascribed to the amelioration of left ventricular (LV) function, which further complicates the understanding of their underlying mechanism.

**Methods:**

This study is a prospective, phase IV, open-label, parallel group, single-center trial conducted in a large tertiary teaching hospital in China. A total of 120 patients with AMI and type 2 diabetes mellitus (T2DM) will be included. Those who received SGLT2 inhibitors are considered as the experimental group, and those taking other antidiabetic agents are considered as the control group. The primary outcome is change in LV end-systolic volume index (LVESVi) measured by cardiac magnetic resonance (CMR) imaging from baseline during 1-year follow-up period. Secondary outcomes include other LV parameters such as LV mass, LV volume, and LV ejection fraction (EF); quality of life and functional capacity such as Kansas City Cardiomyopathy Questionnaire overall summary score (KCCQ-OS) and EuroQol-5 dimension (EQ-5D); biomarkers associated with diagnostic parameters of AMI and possible mechanisms on cardiovascular protection, such as creatine kinase, troponin T (TnT) level, troponin I (TnI) level, soluble suppression of tumorigenicity-2 (sST2), galectin-3 (Gal-3), fibroblast growth factor 21 (FGF21), and microRNA (miRNA) level.

**Discussion:**

This study aims to investigate whether SGLT2 inhibitors could improve LV function by measuring CMR, quality of life, and functional capacity in patients with AMI in real-world settings, providing evidence on the underlying mechanism of SGLT2 inhibitors on cardioprotection.

**Clinical trial registration:**

https://www.chictr.org.cn/showproj.html?proj=173672, identifier ChiCTR2200065792.

## 1 Introduction

Type 2 diabetes mellitus (T2DM) is an independent and strong risk factor for cardiovascular events, exhibiting a double increased susceptibility to cardiovascular diseases (CVDs) when compared to individuals without T2DM (1, 2). Sodium-glucose cotransporter-2 (SGLT2) inhibitors (SGLT2i) are initially used as glucose-lowering agents in T2DM patients with CVD. The emergence of substantial cardiovascular outcome clinical trials investigating SGLT2i, such as empagliflozin (EMPA-REG OUTCOME) (3), dapagliflozin (DECLARE-TIMI 58) (4), canagliflozin (CANVAS Program) (5), ertugliflozin (VERTIS CV) (6), and sotagliflozin (SCORED) (7), has demonstrated the effects of SGLT2i on the reduction of the occurrence of cardiovascular events and hospitalization for heart failure (HF) in T2DM patients with high risks for CVD or chronic kidney disease (CKD). From the angle of underlying mechanisms, long-term exposure to hyperglycemia affected signaling pathways inducing electrical disturbances in cardiomyocytes and mitochondrial dysfunction, prompting elevated reactive oxygen species (ROS) production and even apoptosis (8, 9). Preclinical data demonstrated (10, 11) that SGLT2i inhibit Na^+^/hydrogen exchanger 1 (NHE-1), leading to a reduction of Na^+^ in cardiac cytoplasm whose high expression was considered as a denominator of diabetes and HF and increasing Ca^2+^ level in cardiac mitochondria to improve ATP generation and viability of cardiomyocytes. Another explanation for the benefit in cardiomyocytes is that empagliflozin could reduce Ca^2+^/calmodulin-dependent kinase II (CaMKII) activity in both HF murine and human ventricular cardiomyocytes to improve contractility, since increased CaMKII levels are considered as hallmarks of HF (12). Moreover, several mechanistic investigations (13) also focused on its cardiovascular protective effects encompassing improvement in myocardial efficiency and endothelial function and reduction of oxidative stress, fibrosis, and inflammation of the heart. Thus, the recent guideline strongly recommends patients with T2DM and CVD to receive SGLT2i whether they are treatment-naive or already on metformin (14).

Acute myocardial infarction (AMI) poses a significant threat to CVDs, leading to a high risk of HF occurrence and cardiovascular death (15–17). Although patients receive updated evidence-based therapies early post-infarction, the contemporary rates of adverse cardiovascular outcomes remain high (18, 19). Recent preclinical studies (20, 21) in AMI mice treated with SGLT2i demonstrated new mechanistic insights into the reduction of infarction size and cardiomyocyte apoptosis, while data from clinical trials are still lacking in AMI. Thus far, conflicting outcomes were reported in studies on cardiac function with SGLT2i, likely stemming from factors such as limited sample size, short follow-up periods, and variations in patient characteristics (22–26). In our prior meta-analysis involving 13 trials and involved 1,437 participants, we observed that SGLT2i could improve LV function, and most studies focused on empagliflozin or HF patients (26). In this study, we present a protocol to assess the effects of SGLT2i on cardiac function in AMI patients. Meanwhile, other outcomes, including quality of life, functional capacity, and potential mechanisms, will also be discussed.

## 2 Materials and methods

### 2.1 Study design and setting

This is a prospective, phase IV, open-label, parallel group, single-center trial in patients with T2DM and AMI. The purpose of this trial is to assess the effect of SGLT2i on cardiac structure and cardiac function in these high-risk patients. Furthermore, we will evaluate the quality of life and other dimensions in these high-risk HF populations by questionnaire’s instructions [Kansas City Cardiomyopathy Questionnaire overall summary score (KCCQ-OS) and EuroQol-5 dimension (EQ-5D)]. Finally, biomarkers associated with diagnostic parameters of AMI [creatine kinase, troponin T (TnT), troponin I (TnI)], the potential mechanism on cardiovascular protection associated with the biomarkers [soluble suppression of tumorigenicity-2 (sST2), fibroblast growth factor 21 (FGF21), galectin-3 (Gal-3)], and microRNA (miRNA) level will be explored. The trial is registered by School of Medicine, Shanghai Jiao Tong University (Trial Registration: ChiCTR2200065792).

### 2.2 Inclusion and exclusion criteria

#### 2.2.1 Inclusion

(I) Men and women aged 18–80 years with T2DM and AMI undergoing successful percutaneous coronary intervention (PCI) within 14 days of hospital admission;

(II) treated with at least one antidiabetic drug besides lifestyle interventions;

(III) estimated glomerular filtration rate (eGFR) ≥45 mL/min/1.73 m^2^;

(IV) diagnosed with either ST-elevation myocardial infarction (STEMI) or non-ST elevation myocardial infarction (NSTEMI) according to the Fourth Universal Definition of Myocardial Infarction (27) and T2DM diagnosed based on the guideline for the prevention and treatment of T2DM in China (2020 edition) (28).

(V) Severe myocardial necrosis with a rise in CK >800 U/L and a TnT level or TnI level >10× upper limit of normal (ULN) after AMI (29, 30).

#### 2.2.2 Exclusion

(I) Patients with poor compliance;

(II) History of new-set AMI over 2 months.

(III) eGFR <45 mL/min/1.73 m^2^ or on dialysis (derived using CKD EPI);

(IV) currently pregnant or lactating women.

The detailed eligibility and exclusion criteria are provided in **Table 1**.

**Table 1:** List of inclusion and exclusion criteria.

Inclusion criteria
1. Men and women aged 18–80 years with T2DM and AMI undergoing successful percutaneous coronary intervention (PCI) within 14 days of hospital admission
2. Diagnosed with either ST-elevation myocardial infarction (STEMI) or non-ST elevation myocardial infarction (NSTEMI) according to the Fourth Universal Definition of Myocardial Infarction
3. Severe myocardial necrosis with a rise in creatine kinase >800 U/L and a troponin T (TnT) level or troponin I (TnI) level >10× upper limit of normal (ULN) after AMI
4. Diagnosed with T2DM based on the guideline for the prevention and treatment of type 2 diabetes mellitus in China (2020 edition)
5. eGFR ≥45 mL/min/1.73 m^2^
6. Treated with at least one antidiabetic drug besides lifestyle interventions to lower blood glucose
7. Capable of offering signed informed consent and understanding the protocol
8. Good Adherence: Having good compliance (taking more than 80% of monthly pill count), being able to understand, perform, and agree to the prescriber physician’s instructions
Exclusion criteria
1. Currently pregnant or lactating women
2. History of new-onset AMI over 2 months
3. eGFR <45 mL/min/1.73 m^2^ or on dialysis (derived using CKD EPI)
4. Cognitive impairment and failure to complete scheduled follow-up
5. Any contraindication to CMR procedures

AMI, acute myocardial infarction; STEMI, ST-elevation myocardial infarction; NSTEMI, non-ST elevation myocardial infarction; T2DM, type 2 diabetes mellitus; eGFR, estimated glomerular filtration rate.

### 2.3 Intervention

Patients with T2DM will continue the previous glucose-lowering regimen unless glycemic control deteriorates requiring regimen to be adjusted. Patients who received SGLT2i are considered as SGLT2 inhibitors group, and patients not taking any SGLT2i are considered as non-SGLT2 inhibitors group (control group). Based on the requirement of open-label study, patients will be assigned to a 1-year treatment with either SGLT2i (empagliflozin 10 mg/day or dapagliflozin 10 mg/day or canagliflozin 100 mg/day) or other antidiabetic drugs [insulin, metformin, α-glucosidase inhibitors, sulfonylurea hypoglycemic agents, dipeptidyl peptidase-4 inhibitors (DPP4i), glucagon-like peptide-1 (GLP1) receptor agonists, and so on] (31). Patients without SGLT2i contraindication were prescribed one type of SGLT2i, other patients used other antidiabetic drugs in order to maintain the blood glucose into the target in the hospitalization (fasting blood glucose 7.8–10 mmol/L, postprandial blood glucose 7.8–13.9 mmol/L) (31). Considering the beneficial effects on LV function and high availability in China (26), empagliflozin was recommended first. Participants with contraindications of SGLT2i including those with the presence of ketone bodies or genitourinary infections might be assigned to the control group without SGLT2i. Of note, DPP4i (saxagliptin or sitagliptin) and thiazolidinediones (such as rosiglitazone or pioglitazone) are not preferred in patients with AMI combined with HF, which may worsen HF and cause hospitalization based on the present guideline (32).

**Figure 1:**
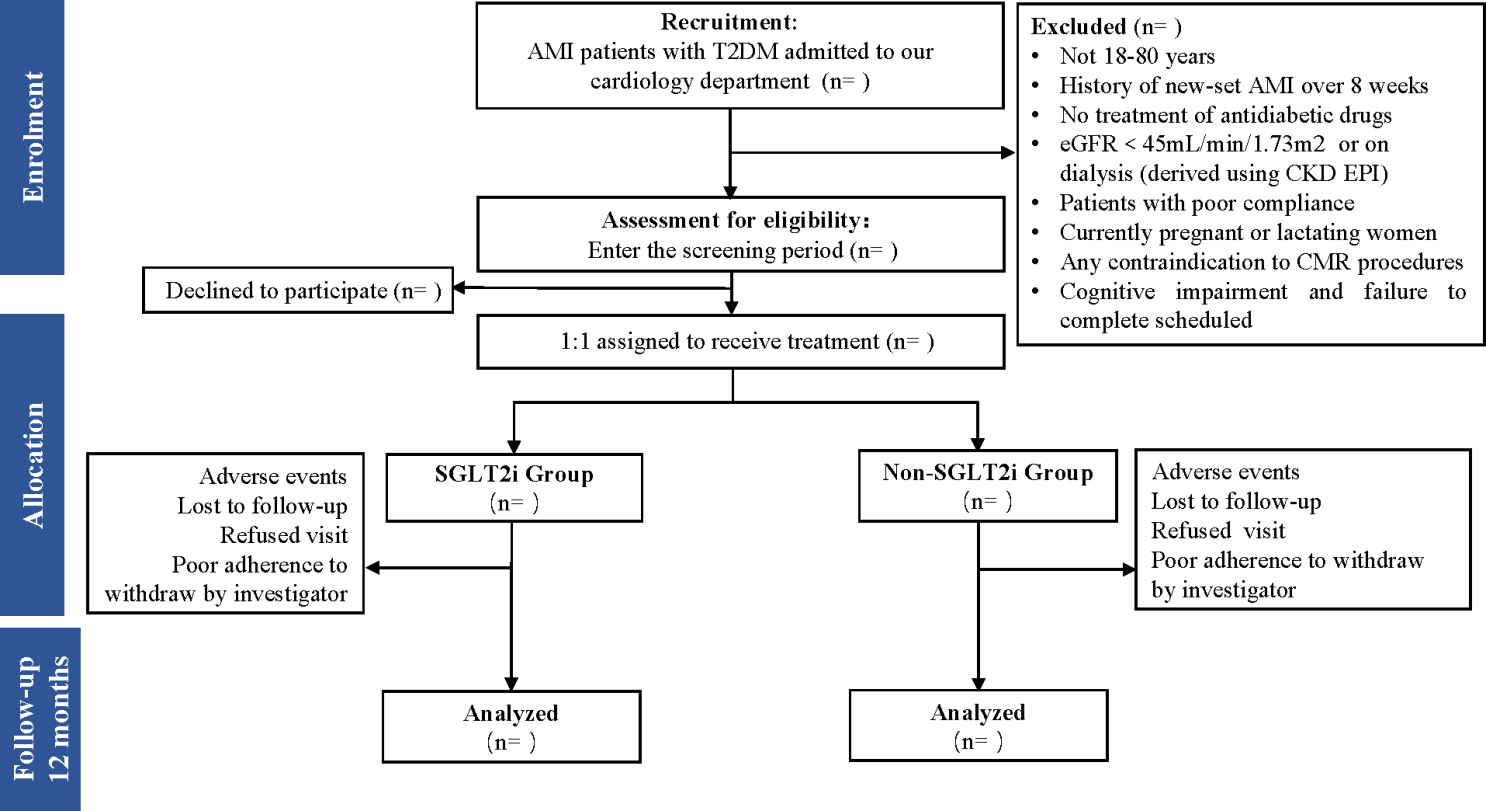
Flow diagram of the study patient disposition throughout the trial. AMI, acute myocardial infarction; T2DM, type 2 diabetes mellitus; eGFR, estimated glomerular filtration rate; SGLT2i, sodium-glucose cotransporter-2 inhibitor.

Patients will participate in three visits (at 3, 6, and 12 months after hospitalization for new-onset AMI) in 1 year (see **Figure 1** for an overview of all visits’ process).

### 2.4 Endpoints

The primary endpoint is the change of cardiac function [LV end-systolic volume index (LVESVi)] measured by cardiac magnetic resonance (CMR) in T2DM patients with AMI. The secondary endpoints are (I) other changes in parameters of cardiac function measured by CMR and echocardiography (LVEF, LV mass, LV volume); (II) change in KCCQ-OS and EQ-5D from baseline; (III) change in biomarkers related to myocardial parietal stress [N-terminal fragment brain natriuretic peptide (NT-pro BNP)], myocyte injury (creatine kinase, TnI, TnT), fibrosis (sST2, Gal-3), inflammation including interleukin-1β (IL-1β), interleukin-6 (IL-6), interleukin-8 (IL-8), interleukin-10 (IL-10), tumor necrosis factor-α (TNF-α), procalcitonin (PCT), C-reactive protein (CRP), and FGF21 in hospital laboratory research, as well as miRNA level. The detailed endpoints are provided in **Table 2**.

**Table 2:** Trial endpoints.

	Outcome measures
Primary endpoint	
Cardiac function in T2DM patient with AMI 12 months after the event	Change in LVESVi from baseline to the study end at 12 months measured by cardiac MRI
Secondary endpoints	
1. Parameters of cardiac structure	Change in LVEF, LV mass, LV volume at 3 months, 6 months, and 12 months after hospitalization for new-onset AMI
2. Biomarkers related to myocardial parietal stress 3. Biomarkers related to AMI 4. Biomarkers related to fibrosis 5. Biomarkers related to inflammation	Change in NT-pro BNP; Change in CK, TnI, TnT; Change in sST2, Gal-3; Change in IL-1β, IL-6, IL-8, IL-10, TNF-α, CRP, and PCT; at 3 months, 6 months, and 12 months after hospitalization for new-onset AMI
6. Questionnaire’s instructions on health status	Change in KCCQ-OS and EQ-5D at 3 months, 6 months, and 12 months after hospitalization for new-onset AMI
Exploratory endpoints on the mechanisms	
To investigate that whether SGLT2i will have an effect on miRNAs and FGF21	Change in miRNAs and FGF21

AMI, acute myocardial infarction; LVESVi, LV end-systolic volume index; MRI, magnetic resonance imaging; eGFR, estimated glomerular filtration rate; SGLT2i, sodium-glucose cotransporter-2 inhibitor; LV, left ventricular; EF, ejection fraction; NT-pro BNP, N-terminal fragment brain natriuretic peptides; CK, creatine kinase; TnI, Troponin I; TnT, Troponin T; sST2, soluble suppression of tumorigenicity-2; Gal-3, galectin-3; KCCQ-OS, Kansas City Cardiomyopathy Questionnaire overall summary score; EQ-5D, EuroQol- 5 dimension; FGF21, fibroblast growth factor 21.

### 2.5 CMR and analysis

Contrast-enhanced CMR will be performed on a clinical 3.0-Tesla scanner (Achieva TX, Philips Healthcare, Best, Netherlands) within 14 days after the first PCI and at 1-year follow-up. All images will be obtained in breath-hold with a default field of view of 350 × 350 mm^2^. An experienced reader blinded to clinical data will analyze the CMR image with commercially available software (QMassx MR 7.5, Medis Medical Imaging, Leiden, Netherlands). The endocardial contour of the LV will be delineated excluding trabeculations and LV papillary muscles. In cases with significant discrepancy, the contours will be reviewed and corrected by a consensus group. Quantitative detection of LV volume and LVEF will be calculated based on short-axis slices of cine images covering the whole heart at end systole and end diastole, respectively. The extent of both infarction and microvascular obstruction will be semiquantified as a percentage of LV myocardial mass (% LVM).

### 2.6 Questionnaire’s instructions

KCCQ-OS is designed for patients with HF to evaluate the frequency of HF symptom, physical limitation, quality of life, and social limitation (33, 34). EQ-5D is an instrument to evaluate the quality of life in terms of five dimensions including mobility, self-care, activities, anxiety/depression, and pain/discomfort (35). Both full scores are ranging from 0 to 100, with higher scores reflecting better health status. The KCCQ and EQ-5D questionnaires are documented in **Supplementary Table S1** and **Supplementary Table S2**, respectively.

### 2.7 Biomarkers of cardiac function

#### 2.7.1 Blood samples

Blood samples for routine biomarkers (NT-pro BNP, creatine kinase, TnI, TnT, sST2, IL-1β, IL-6, IL-8, IL-10, TNF-α, CRP, PCT, Gal-3) will be collected and measured by the hospital laboratory service from the eligible patients within 14 days after AMI incidence and at 1-year follow-up. A total of approximately 8 mL of blood samples will be drawn from the antecubital vein into two vacuum blood collection tubes at two time points: on the first 14 days of AMI (admission) and at 1 year following AMI. To obtain plasma, 4 mL of blood sample with anticoagulants (EDTA) will be centrifuged at 3,000 rpm for 10 min at 4°C, then the yellow cell-free supernatant will be collected and frozen in RNase-free Cryo-Tubes under -80°C until further processing. To obtain serum, the tubes without anticoagulants within 4 mL of peripheral blood samples are let stand at a 45- to 60-degree angle under 4°C for at least 1 h to promote coagulation. Then, the blood is centrifuged within 10 min of withdrawal at 3,000 rpm for 10 min to obtain the serum, which will be separated into RNase-free tubes. The handled samples are stored under -80°C to be used further for the miRNAs and FGF21 detector.

#### 2.7.2 Analysis of FGF21

The concentration of serum FGF21 is measured by a commercially quantitative human ELISA kit (DF2100, R&D Systems, Minneapolis, MN, USA) according to the manufacturer’s instructions. Absorbance at 450 nm is performed using a microplate reader (BioTek, Winooski, VT, USA). According to the manufacturer’s information, the inter-assay and intra-assay coefficients of variation were 2.9%–3.9% and 5.2%–10.9%, respectively.

#### 2.7.3 Analysis of miRNA

Total RNA is extracted from the handled serum *via* standard procedures using DNase I RNase-free (Takara) to remove genomic DNA according to the manufacturer’s instruction (Invitrogen). The purity and concentration of our RNA are measured using ND-2000 (NanoDrop Technologies). RNA integrity is evaluated by 2100 Bioanalyzer (Agilent Technologies, Santa Clara, CA, USA). First-strand cDNA is synthesized using M-MuLV Reverse Transcriptase (RNase H–). Amplification and detection by polymerase chain reaction (PCR) are performed using LongAmp Taq 2X Master Mix, SR Primer. The expression of miRNA is assessed by quantitative real-time PCR and calculated according to the transcripts per million reads (TPM) method. Significant differently expressed (DE) miRNAs are extracted with |log2 fold change (FC)| >1 and False Discovery Rate (FDR) <0.05 by DEseq2.

### 2.8 Study statistics

#### 2.8.1 Sample size calculation

The sample size of this trial is calculated based on the primary outcome—the change in LVESVi. In a recent randomized controlled trial (SUGAR-DM-HF), the mean ± standard deviation (SD) of change in LVESVi in the experimental group and control group was -7.9 ± 11.8 mL/m^2^ and -1.5 ± 11.3 mL/m^2^, respectively (24). These data were for an 80% power at a 2.5% significance level [α = 0.025] with a 1% margin of error. Sample size was calculated to be 108 patients randomly assigned 1:1 to empagliflozin 10 mg once daily or placebo (24). Considering a 10% dropout in our previous study due to the presence of dressing change or medication nonadherence, a sample size of 120 subjects (two groups of 60) is necessary.

#### 2.8.2 Statistical analysis

Continuous variables will be described as mean with SD and compared by unpaired Student’s t-tests or Mann–Whitney U tests. Categorical variables of baseline characteristics will be summarized using count statistics, presented as percentage n (%) as appropriate. Chi-square test will be used when sample size is larger than 40; Fisher’s exact test will be used when sample size is smaller than 40. The results with p values <0.1 in univariate regression or considered meaningful in clinical practice will be further analyzed using multivariate regression to explore the effects of SGLT2i on LV function. All analyses will be conducted using the SPSS software, version 22.0 (SPSS Inc., Chicago, IL, USA).

## 3 Discussion

Several studies (3–7) have demonstrated the cardiovascular protective effects of SGLT2i in T2DM patients with high cardiovascular risks. However, inconsistent results on LV functions were shown in clinical trials (22–26) related to specific gliflozin. Thus, in this study, we will concentrate on the influence of SGLT2i on cardiac function.

Limited by a small sample size (only 28 patients per treatment group) and mild severity of HF, the first explorative clinical study (REFORM) failed to observe the effect of dapagliflozin on LVESVi or any other parameter of LV remodeling in participants with symptomatic HF and T2DM (22). Similarly, the EMPA-HEART CardioLink-6 trial showed that empagliflozin had no effect on LVESVi or LV end-diastolic volume index (LVEDVi) in patients with T2DM and coronary artery disease (CAD) (23), with a short follow-up period (only 6 months) and a small portion (8%) of participants having HF. In contrast to the aforementioned studies, SUGAR-DM-HF exhibited that empagliflozin could reduce both LVESVi and LVEDVi in patients with T2DM and reduced ejection fraction (HFrEF) with the mean LVEF (32.5%) for only 9 months (24). The similar positive results were also revealed in EMPA-TROPISM in nondiabetic patients with HFrEF (25). The distinct participant characteristics, disease severity, and sample size are possible explanations for conflicting findings on the relationship between the use of SGLT2i and LV function. Alternatively, we proposed that the background treatment including angiotensin-converting enzyme inhibitor/angiotensin receptor antagonist (empagliflozin vs. dapagliflozin: 82.1% vs. 78.7%) and β-blockers (empagliflozin vs. dapagliflozin: 89.7% vs. 45.0%) was partly attributed to the inconsistent study results (26). We found that the reported improvement of LV function was mainly derived from HF population with lower LVEF or receiving empagliflozin (26).

CMR has emerged as the gold standard for quantifying cardiac function and possess higher reproducibility than echocardiography (36). Recent studies demonstrated that CMR had the potential capability to elucidate the detailed changes in ventricular structure especially in intracellular or extracellular compartments (26, 36). Given that, we performed CMR in addition to echocardiography to acquire more precise images of LV structure, with a specific focus on changes in LVESVi.

The target population in this study are patients with T2DM and AMI. A large cohort study (n = 2,596) manifested that almost 70% of AMI participants developed HF within 7.6 years, which was associated with LV remodeling (37, 38). Thus, early intervention to prevent LV remodeling and post-AMI heart failure is crucial. Due to a growing body of evidence on SGLT2i in a CVD population (19), it is reasonable to investigate whether early initiation of SGLT2i in patients with AMI improves cardiovascular outcomes. In the EMPA-REG OUTCOME trial, empagliflozin decreased cardiovascular mortality and HF hospitalization in patients with T2DM and a history of MI (3). At present, eight clinical investigations related to SGLT2i have been conducted in AMI patients with DM (9, 39, 40). Notably, three studies, specifically the EMMY study (41), SGLT2-I AMI PROTECT (39, 40), and EMBODY (42) study, have effectively disseminated their findings. The EMMY study revealed that the changes in LVESV and LVEDV were lower by 7.5 mL (p = 0.0003) and 9.7 mL (p = 0.0015), respectively, in the empagliflozin group compared to the placebo group, and a significant reduction in NT-pro BNP levels was observed in the empagliflozin group (41). An observational registry SGLT2-I AMI PROTECT exhibited that those receiving SGLT2-I can significantly reduce inflammatory response and infarct size compared to those receiving other oral antidiabetic agents, and the use of SGLT2-I showed a lower risk of adverse cardiovascular outcomes (39, 40). The EMBODY study demonstrated that early SGLT2i administration might be effective in improving cardiac nerve activity without any adverse events (42). So far, the remaining five studies (EMPACT-MI, EMPRESS MI, DAPA-MI, NCT03658031, and NCT03591991) have not yet been published (9, 43). These studies did not explore the cellular-level mechanisms throughout their investigations, while our research endeavors to study the impact of SGLT2i on the structural aspects of the cardiac domain within the Asian population. To achieve this, we employ magnetic resonance imaging and blood work on its potential mechanisms underlying the favorable action on cardioprotection function. Additionally, such high-risk population with diabetes and AMI are commonly paid more attention on improvement in quality of life (14). A recent prospective study has provided compelling evidence suggesting the beneficial effect of empagliflozin on cognitive and physical impairment in frail older adults with T2DM and preserved ejection fraction (HFpEF) (44). This finding has sparked our interest in investigating whether the results are similar to those of a population with T2DM and AMI. KCCQ-OS is initially designed for patients with HF to evaluate symptom frequency, physical limitation, quality of life score, and social limitation (33). A recent cohort study included HFrEF patients exhibited that KCCQ-OS is more sensitive in health status over time than New York Heart Association (NYHA) class considering as a cornerstone for quantifying the health status of HF population (34). It is unknown whether KCCQ-OS is applicable to AMI population with high risks of HF. Moreover, we will also use EQ-5D to evaluate the quality of life in terms of five dimensions including mobility, self-care, activities, anxiety/depression, and pain/discomfort (35). Both full scores are ranging from 0 to 100, with higher scores reflecting better health status.

The underlying mechanisms of cardioprotection effects of SGLT2i are not completely understood, since only a few preclinical trials revealed that AMI mice treated with empagliflozin could improve LV remodeling by inhibiting cardiomyocyte apoptosis and altering myocardial substrate utilization (20, 21). Thus, we hope to explore the mechanistic benefits of SGLT2i in patients with AMI and provide evidence for early initiation following AMI. miRNAs, as noncoding RNAs, can regulate some posttranscriptional genes associated with the occurrence of AMI and are considered as early prediction markers with higher sensitivity than wildly acknowledged biomarkers (such as NT-pro BNP) (45). Additionally, a recent clinical study found that miR-29b levels were associated with changes of LVEDV (p < 0.05) measured by CMR over time after AMI (46). The latest research demonstrated that specific miRNAs (miR-126, miR-342-3p, miR-638, miR-21, and miR-92) were significantly regulated in T2DM and HFpEF patients and in response to empagliflozin (47). Current review suggested that four miRNAs (miR-29a, miR-29b, miR-150, and miR-30a-5p) may be prognostic markers in the patient’s clinical status associated with post-AMI LV dysfunction (48). Therefore, the involving panel of candidate miRNAs are measured to understand pathophysiological effects of SGLT2i and its correlation with CMR, as well as alterations in creatine kinase, TnI, or TnT level—diagnostic parameters indicative of AMI. The other exploratory mechanism is by changing FGF21 level. In recent clinical trials, FGF21 was proven to be an independent predictor of coronary heart disease in patients with T2DM and increased cardiovascular risks and was used as a novel biomarker to predict major adverse cardiovascular events (MACEs) in patients with STEMI after PCI (49–51). Our study is designed to test the hypothesis that treatment with SGLT2i changes FGF21 levels in patients with AMI.

The main strengths of this study are that we investigate the effects of SGLT2i on LV function using CMR technology in real-world scenario. Moreover, it explores the potential underlying mechanism that preserves ventricular function in these high-risk patients. Furthermore, the assessment of quality of life in AMI patients at high risk for HF on SGLT2i is conducted using KCCQ and EQ-5D.

However, it is important to acknowledge certain limitations of this study. This trial is a parallel group and single-center research that recruits patients with T2DM and severe AMI with complex conditions such as HF or CKD. Thus, whether these findings can speculate to other more uncomplicated patients needs to be further studied. To enhance the credibility of the results, the study employs univariate and multivariate analyses to migrate the possible influences.

## 4 Conclusion

This is a prospective, phase IV, open-label, parallel group, single-center trial in patients with T2DM and AMI. It will investigate whether SGLT2i could improve LV function by measuring CMR, quality of life, and functional capacity in patients with AMI in real-world settings, providing evidence on the underlying mechanism of SGLT2i on cardioprotection.

## Ethics statement

Written informed consent was obtained from the individual(s) for the publication of any potentially identifiable images or data included in this article.

## Author contributions

JP and LS are the guarantors of the entire article. M-JC, F-HS, and HG drafted the article. B-BY, X-CM, CZ, LX, and Y-HJ contributed to the study conception and design, critical revision of the article for important intellectual content, and final approval of the version to be published. All authors discussed to help develop the protocol and read and approved the final article. All authors contributed to the article and approved the submitted version.
