# Supplementary material for: Influence and mechanism of sodium-glucose cotransporter-2 inhibitors on the cardiac function: study protocol for a prospective cohort study
Source: Front Endocrinol (Lausanne). 2023 Jul 19;14:1199960. doi: 10.3389/fendo.2023.1199960 (PMC10395085; doi:10.3389/fendo.2023.1199960)
Supplement: Supplementary file 1 [file Table_1.docx]

**Table S1. Kansas City Cardiomyopathy Questionnaire**

The following questions refer to your heart failure and how it may affect your life. Please read and complete the following questions. There are no right or wrong answers. Please mark the answer that best applies to you.

1. Heart failure affects different people in different ways. Some feel shortness of breath while others feel fatigue. Please indicate how much you are limited by heart failure (shortness of breath or fatigue) in your ability to do the following activities over the past 2 weeks.

| Activity | Extreme | Quite a bit | Moderate | Slight | Not at all | Others ^a^ |
| --- | --- | --- | --- | --- | --- | --- |
| Dress yourself | A | B | C | E | D | F |
| Shower/Bathe | A | B | C | D | E | F |
| Walk 1 block on level ground | A | B | C | D | E | F |
| Do yardwork, housework… | A | B | C | D | E | F |
| Climb a flight of stairs without stopping | A | B | C | D | E | F |
| Hurry or jog | A | B | C | D | E | F |

^a^ Nonparticipation or for other reasons

1. Compared with 2 weeks ago, have your symptoms of heart failure (shortness of breath, fatigue or ankle swelling) changed? My symptoms of heart failure have become...
2. Much worse
3. Slightly worse
4. Not changed
5. Slightly better
6. Much better
7. I’ve had no symptoms over the last 2 weeks
8. Over the past 2 weeks, how many times did you have swelling in your feet, ankles or legs when you woke up in the morning?
9. Every morning
10. 3 or more times a week, but not every day
11. 1–2 times a week
12. Less than once a week
13. Never over the past 2 weeks
14. Over the past 2 weeks, how much has swelling in your feet, ankles or legs bothered you? It has been . . .
15. Extremely bothersome
16. Quite a bit bothersome
17. Moderately bothersome
18. Slightly bothersome
19. Not at all bothersome
20. I’ve had no swelling
21. Over the past 2 weeks, on average, how many times has fatigue limited your ability to do what you want?
22. All the time
23. Several times per day
24. At least once a day
25. 3 or more times per week but not every day
26. 1–2 times per week
27. Less than once a week
28. Never over the past 2 weeks
29. Over the past 2 weeks, how much has your fatigue bothered you? It has been . . .
30. Extremely bothersome
31. Quite a bit bothersome
32. Moderately bothersome
33. Slightly bothersome
34. Not at all bothersome
35. I’ve had no fatigue
36. Over the past 2 weeks, on average, how many times has shortness of breath limited your ability to do what you wanted?
37. All the time
38. Several times per day
39. At least once a day
40. 3 or more times per week but not every day
41. 1–2 times per week
42. Less than once a week
43. Never over the past 2 weeks
44. Over the past 2 weeks, how much has your shortness of breath bothered you? It has been . . .
45. Extremely bothersome
46. Quite a bit bothersome
47. Moderately bothersome
48. Slightly bothersome
49. Not at all bothersome
50. I’ve had no shortness of breath
51. Over the past 2 weeks, on average, how many times have you been forced to sleep sitting up in a chair or with at least 3 pillows to prop you up because of shortness of breath?
52. Every night
53. 3 or more times a week, but not every day
54. 1–2 times a week
55. Less than once a week
56. Never over the past 2 weeks
57. Heart failure symptoms can worsen for a number of reasons. How sure are you that you know what to do, or whom to call, if your heart failure gets worse?
58. Not at all sure
59. Not very sure
60. Somewhat sure
61. Mostly sure
62. Completely sure
63. How well do you understand what things you are able to do to keep your heart failure symptoms from getting worse? (for example, weighing yourself, eating a low salt diet, etc.)
64. Do not understand at all
65. Do not understand very well
66. Somewhat understand
67. Mostly understand
68. Completely understand
69. Over the past 2 weeks, how much has your heart failure limited your enjoyment of life?
70. It has extremely limited my enjoyment of life
71. It has limited my enjoyment of life quite a bit
72. It has moderately limited my enjoyment of life
73. It has slightly limited my enjoyment of life
74. It has not limited my enjoyment of life at all
75. If you had to spend the rest of your life with your heart failure the way it is right now, how would you feel about this?
76. Not at all satisfied
77. Mostly dissatisfied
78. Somewhat satisfied
79. Mostly satisfied
80. Completely satisfied
81. Over the past 2 weeks, how often have you felt discouraged or down in the dumps because of your heart failure?
82. I felt that way all the time
83. I felt that way most of the time
84. I occasionally felt that way
85. I rarely felt that way
86. I never felt that way
87. How much does your heart failure affect your lifestyle? Please indicate how your heart failure may have limited your participation in the following activities over the past 2 weeks.

| Activity | Severe | Quite a bit | Moderate | Slight | Not at all | Others ^a^ |
| --- | --- | --- | --- | --- | --- | --- |
| Hobbies, recreational activities | A | B | C | E | D | F |
| Worker do household chores | A | B | C | D | E | F |
| Visit family or friends out of your home | A | B | C | D | E | F |
| Intimate relationships with lovers | A | B | C | D | E | F |

^a^ Nonparticipation or for other reasons
