# Supplementary material for: Influence and mechanism of sodium-glucose cotransporter-2 inhibitors on the cardiac function: study protocol for a prospective cohort study
Source: Front Endocrinol (Lausanne). 2023 Jul 19;14:1199960. doi: 10.3389/fendo.2023.1199960 (PMC10395085; doi:10.3389/fendo.2023.1199960)

**Table S2. The European Quality of Life-5 Dimensions Questionnaire**

By placing a tick in one box in each group below, please indicate which statements best describe your own health state today.

**1. Mobility**

◻ I have no problems in walking about

◻ I have some problems in walking about

◻ I am confined to bed

**2. Self-care**

◻ I have no problems with self-care

◻ I have some problems washing or dressing myself

◻ I am unable to wash or dress myself

**3. Usual activities** (e.g. work, study, housework, family or leisure activities)

◻ I have no problems with performing my usual activities

◻ I have some problems with performing my usual activities

◻ I am unable to perform my usual activities

**4. Pain/discomfort**

◻ I have no pain or discomfort

◻ I have moderate pain or discomfort

◻ I have extreme pain or discomfort

**5. Anxiety/depression**

◻ I am not anxious or depressed

◻ I am moderately anxious or depressed

◻ I am extremely anxious or depressed

To help people say how good or bad a health state is, we have drawn a scale (rather like a thermometer) on which the best state you can imagine is marked 100 and the worst state you can imagine is marked 0. We would like you to indicate on this scale how good or bad your own health is today, in your opinion. Please do this by drawing a line from the box below to whichever point on the scale indicates how good or bad your health state is today.


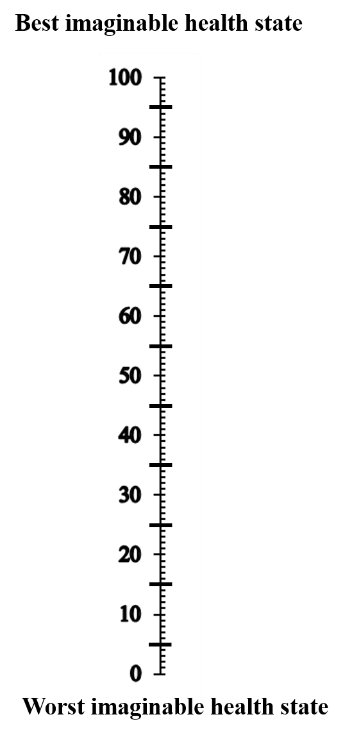

Supplement: Supplementary file 2 [file Table_2.docx]
